# Fact or hypothesis: concomitant immunity in taeniid cestode infections

**DOI:** 10.1111/j.1365-3024.2010.01227.x

**Published:** 2010-08

**Authors:** M W LIGHTOWLERS

**Affiliations:** Faculty of Veterinary Science, The University of MelbourneWerribee, Victoria, Australia

**Keywords:** *Cestode*, Echinococcus, *immunity*, Taenia, *vaccination*

## Abstract

Sustained research efforts over the last 50 years have revealed a considerable amount of information about immunity to taeniid cestode infections in the parasites’ intermediate hosts. As a product of this research, a series of effective recombinant vaccines have been developed which have no parallel in any other group of parasitic organisms. There are, however, many important aspects relating to immunity that remain to be elucidated. Some concepts have come to be firmly held as facts and yet the supportive data are either conflicting or unconfirmed. This review considers the phenomenon of immunity to re-infection with taeniid cestodes in their intermediate hosts, examining carefully the nature of the evidence that is available to support conclusions that have been drawn in this area.

## Introduction

Taeniid cestodes are unique among the helminth parasites in that protective immune responses can be readily demonstrated in the parasites’ intermediate hosts. In retrospect, it seems unsurprising that one of the first clear demonstrations of the induction of immunity to a metazoan parasite was achieved with a taeniid cestode (1). A great deal is now understood about the nature of the protective immune responses to taeniid cestodes, stemming initially from the work of Harry M. Miller in the 1930s and developed through seminal contributions by Michael A. Gemmell, David D. Heath, Jeffrey F. Williams, Graham F. Mitchell and Michael D. Rickard (2). An understanding of the nature of the host-protective responses (3) has been harnessed over the past 20 years to develop extraordinarily effective recombinant vaccines. These seem likely to play a future role as new tools for the control of hydatid disease and cysticercosis caused by *Taenia solium* (4,5).

Although much is understood about the nature of the protective immune responses to taeniid cestodes, many aspects still remain to be clarified and require further investigation to confirm concepts that have come to be considered as being well-established facts, while the published data may be equivocal. Here, the concept of immunity to re-infection with taeniid cestode parasites in their intermediate hosts is examined with a view to dissecting aspects for which there is good, reproducible evidence and differentiating these from aspects which would be better regarded as hypotheses in need of further experimental assessment.

## Concomitant immunity

One of the hallmarks of the immunology of taeniid cestode infection in their intermediate hosts is that infected hosts are immune to re-infection. This situation is sometimes referred to as concomitant immunity, a term adopted in the 1980s from the field of tumour immunology (6). In this situation, an infected animal is immune to re-infection, while at the same time parasites from the initial infection remain unaffected.

Immunity to re-infection has been demonstrated experimentally in many taeniid parasite/host systems, however, it seems likely that this immunity is not associated with previous infection *per se* but rather to previous exposure to host-protective antigens unique to the oncosphere and early developing larvae. To date few experiments have provided data that can be used to directly support this hypothesis. Data that are available suggest that immunity to ‘re-infection’ may arise in a situation where a host is exposed to taeniid eggs even if this does not lead to the establishment of an on-going viable infection and that immunity wanes in the continued presence of viable metacestodes. Hence, immunity to re-infection in the intermediate hosts of taeniid cestodes may be a reflection of the host’s exposure to antigens associated with the early invading parasite, irrespective of whether a (continuing) infection is established by the initial exposure to infective parasites.

Harry M. Miller (7) credits Vogel (8) with the first description of immunity to superinfection in the intermediate hosts of *Taenia taeniaeformis*, however, it was Miller himself who clearly demonstrated the phenomenon and defined many of its characteristics. While working on studies investigating whether it was possible to stimulate artificial immunity to an experimental challenge infection with eggs of *T. taeniaeformis* in rats, Miller (7,9) noticed that occasionally his experimental rats failed to become infected after he administered an oral challenge infection with eggs. At post-mortem, these animals were found to harbour large, mature strobilocerci of *T. taeniaeformis,* indicating that the animals had been exposed to infection with the parasite while they were with his animal supplier. Miller undertook experiments to test the hypothesis that infected animals were immune to re-infection and confirmed this unequivocally (7). Naïve rats also could be protected against infection with *T. taeniaeformis* by injecting serum collected from infected animals (10). Recipients of immune serum were only protected if the serum was given prior to 8 days after the initiation of an infection (11), potency of the serum in transferring passive protection was related to the degree of infection seen in the serum donors (12) and the effectiveness of the serum to transfer protection persisted in donors for at least 2 months after the removal of the larvae from the serum donors via laparotomy (13). Resistance to superinfection was subsequently shown to occur in many different hosts of many species of cestode [see reviews by Lloyd (14), Williams (15) and Rickard and Williams (3)]. Michael Gemmell elevated experiments on immunity to re-infection with taeniid cestodes in sheep to something of an art form. Extending the initial discovery by Froyd and Round (16) that taeniid cestodes would develop at an aberrant tissue site in the host following the injection of activated oncospheres, Gemmell applied this technique to differentiate between parasites arising from a primary infection and those arising from a secondary infection. He demonstrated high levels of protection following an initial exposure to parasites of the homologous species and partial protection when sheep were challenged with a heterologous species of taeniid cestode (17–19).

We can deduce from this information that concomitant immunity can certainly be demonstrated in the intermediate hosts of many species of taeniid cestode. What is not so clear are the parameters surrounding this phenomenon, particularly those that would impact on the phenomenon in naturally infected animals. This is not something of academic interest alone. Computer models are being adopted to assist with predicting the impact of various disease control options for hydatid disease (20–22) and cysticercosis (20,23,24). One of the parameters incorporated into such models is the impact of immunity on the accumulation of an increased parasite burden in older animals as a result of re-exposure. While the evidence is clear that experimental exposure of hosts to infection leads to immunity to a subsequent experimental challenge infection, it is not clear what *level* of initial infection leads to immunity nor the duration that immunity persists. It is also not clear whether the continued persistence of viable parasites from an initial infection maintains immunity to re-infection.

Two ‘phases’ of resistance to infection with taeniid metacestodes are recognized: the pre-encystment phase and the post-encystment phase. This understanding arose initially from experiments on *T. taeniaeformis* in rats where ‘early’ and ‘late’ immunity were recognized and concluded to operate independently (25,26). In sheep, Gemmell (17,18) considered the early phase of immunity to be acting at the level of the intestine (18), making this differentiation on the basis of whether there were no detectable lesions from parasites in a challenge infection (early, intestinal or pre-encystment immunity) as distinct from there being nonviable lesions at the parasites’ site of election (late or post-encystment immunity). Rickard and Williams (3) made the important observation that both of these forms of immunity are acting on the *establishing* parasites. While the phenomenon has been confirmed more recently (27) the immunological basis for the distinction between early and late immunity remains unclear and it is possible that the distinction relates only to the degree of damage inflicted on the early developing parasite, with lesser damage allowing the parasites to survive long enough to create a macroscopically visible lesion (3). A role for immune mechanisms acting within the gut is also possible and could contribute to ‘early’ immunity. Gemmell’s view that the absence of a lesion was associated with immunity acting at the intestinal level does not necessarily follow because the death of a 25-μm oncosphere in the tissues would be unlikely to lead to a macroscopically visible lesion in the tissues which could be found some weeks later when the animals were necropsied. This has been confirmed for *T. taeniaeformis* where the parasites have been shown to reach the liver in passively immunized hosts (28) but are rapidly killed there; such animals typically show no trace of any liver lesions when necropsied several weeks after the challenge infection (10,26,29–31).

The identification of highly protective recombinant antigens from several taeniid species provides some insight into the susceptibility of different lifecycle stages to immunological attack and the ability of different lifecycle stages to maintain immunity by evoking immune responses to these antigens in infected animals. All host-protective antigens that have been defined have been found to be restricted to expression in the oncosphere or very early developmental phases of the parasites (32). Hence, immune responses to these antigens would be expected to have no effect on mature metacestodes and mature metacestodes could not maintain protection in infected animals by continuing to stimulate immune responses to these antigens. There is ample evidence to indicate that extracts from metacestodes contain host-protective antigens [see (1), for example], and immunization with these antigens has its effect against a challenge infection in a similar way to the effects of vaccinating with oncosphere antigens i.e against the oncosphere or early developing (immature) metacestode. While immunization with metacestode antigens can have host-protective effects, it is unclear whether the existence of mature living metacestodes in a host is sufficient to maintain concomitant immunity.

The question remains, not whether it is possible to demonstrate concomitant immunity but whether concomitant immunity is associated with prior infection *per se* and also how much ‘infection’ leads to immunity. Is infection with a single parasite sufficient to establish immunity or to maintain immunity? Knowledge in these areas would be useful to provide accurate information for use in mathematically modelling cestode infections in conditions of different disease endemicity. However, there is very little data on these subjects and what there is suggests that concomitant immunity in cestode infections *may* be more a laboratory artefact than a practical reality in many naturally infected hosts.

There are few data available on what the minimum number of parasites is that a taeniid host must be exposed to generate immunity to further infection. It is important to make a distinction between the number of parasites present in the tissues of an infected host from the number of oncospheres that an animal has been exposed to. Typically, a minority of the parasites that are given in an experimental infection actually establish in a naïve, susceptible host. What happens to the other oncospheres that do not successfully establish is not known, nor is it known whether these oncospheres have the potential to contribute to the stimulation of a protective anti-oncosphere immune response. No experiments have been performed examining immunity arising following infection with a single egg. Miller’s observations that some of the rats supplied to him had a single mature metacestode and were refractory to his experimental infection (7,9), suggests that perhaps only a single metacestode of this species may be sufficient to stimulate and maintain concomitant immunity. Miller noted that often these same rats showed nonviable liver lesions indicating that the animals had indeed been exposed to more than a single oncosphere. The experiments of Miller (33) and Musoke and Williams (34) on transplantation of *T. taeniaeformis* metacestodes may be informative in this respect. Rats can be protected against a challenge infection by surgical implantation of mature cysticerci intraperitoneally. Transplantation of both live cysticerci as well as the same number of nonviable cysticerci (killed by freeze/thawing prior to transplantation) induced protection, although the level of protection was superior in the case of live cysticerci (34). It is difficult to interpret these studies as being the equivalent to the presence of a small number of mature parasites in the animals because the parasites were placed free in the peritoneal cavity rather than being enclosed within the capsule which normally surrounds the parasite in the liver tissue. Also the value of this as a model for other *Taenia* species in this respect can be questioned because the presence of a single large strobilocercus in a small host animal would be the weight equivalent of many thousands of cysticerci of *Taenia ovis*, *Taenia solium* or *Taenia saginata* in sheep, pigs or cattle, respectively. However, Miller’s work with the *T. taeniaeformis*/rat model does provide further clues as to the level of infection required to induce concomitant immunity. Miller and Gardiner (12) found that serum taken from rats with light, long-standing viable infections were either unable to transfer protection to naïve recipients or were poorly protective in comparison to serum taken from donors within weeks of an experimental infection; serum taken from rats that developed no viable parasites was, nevertheless, able to transfer protection and serum from donors with only light infections were substantially less effective in transferring protection than was serum from more heavily infected animals. Kerr (35) also found that rabbits which failed to develop viable parasites from a primary infection with *Taenia pisiformis* were nevertheless immune, or relatively immune, to superinfection.

Other data that pertains to the extent and duration of concomitant immunity comes from the work of Heath and his colleagues with *T. pisiformis* in rabbits and Gemmell and his colleagues with *Taenia hydatigena* in sheep. Heath and Chevis (36) undertook an experiment specifically to determine the duration of concomitant immunity arising after an initial experimental infection with *T. pisiformis* in rabbits and also to determine whether maintenance of this immunity was dependent upon the continued presence of viable parasites from the initial infection. Three groups of 24 rabbits were used, two groups being given an infection with 1000 *T. pisiformis* eggs. One group of infected animals was administered mebendazole between 6–8 weeks post-infection sufficient to kill all the metacestodes. From 9 weeks post-infection and for a further 11 months, two animals from each group were given a challenge infection with *T. pisiformis* eggs and these animals necropsied at a time which allowed differentiation of parasites from the recent infection (migrating in the liver) from those which established from the initial infection (in the peritoneal cavity). Although a small number of the animals which had received the initial infection did show the development of some larvae from the secondary infection after a period of 5 months, the majority remained immune to superinfection at least until approximately 13 months after the initial infection. This was the case both for the animals which maintained viable parasites from the initial infection as well as animals in which all the parasites from the initial infection had been killed by drug treatment 6–8 weeks post-infection.

As mentioned previously, we now know that some or all of the anti-oncosphere immune responses are elicited by antigens that are uniquely expressed by the oncosphere. In the experiments undertaken by Heath and Chevis (36), we are unable to differentiate whether the immunity that persisted for 14 months after the initial infection was the result of the exposure to oncosphere antigens or whether immunity could have been maintained for the lifetime of the infected rabbits as a result of the continued presence of viable metacestodes. It would have been interesting had the animals in this experiment been challenged at longer intervals sufficient to differentiate more effectively between waning anti-oncosphere responses and protective responses maintained by the metacestodes. Heath (37) surgically transplanted *T. pisiformis* larvae of different stages of maturity intraperitoneally into naïve rabbits and noted that larvae up to the age of 15 days were able to confer absolute resistance to a challenge infection. However, after this time the ability of more mature larvae to stimulate protection waned and implantation of mature cysticerci failed to stimulate any protection. This study provides the most convincing evidence that infection with taeniid cestodes *per se* does not lead to a state of concomitant immunity. Rather, exposure to the early developing parasite stimulates a transient protection against the survival of parasites from a subsequent exposure to eggs.

It could be argued that even brief exposure of a host to the early stages of parasite development, even if this does not lead to a mature, continuing infection, is sufficient to come under the definition of ‘prior infection’. This issue is not semantic. The following would have implications for understanding the extent to which immunity to re-infection impacts on the natural regulation in transmission of taeniid cestodes: duration of protection following an initial infection; whether protection occurs in all infected hosts or whether there is a threshold of initial exposure below which no effective immunity to re-infection occurs and whether natural levels of exposure to taeniid eggs in an endemic or hyperendemic environment is sufficient to maintain immunity. Unfortunately, there is very little direct, good quality evidence around any of these important issues.

The bulk of available evidence suggests that experimental exposure of the intermediate hosts of taeniid cestodes to parasite eggs, even if that leads to the early stage of host invasion but not persisting infections, nevertheless can induce protection against a subsequent challenge infection. Evidence from *T. taeniaeformis* in rats has been referred to above and Dow *et al.* (38) and Urquhart *et al.* (39) induced protection in mice and cattle following their exposure to radiation attenuated eggs of *T. taeniaeformis* and *T. saginata*, respectively. One dissenting finding is that of Coman and Rickard (40) who found that rabbits exposed to ‘senescent’ eggs of *T. pisiformis,* which were able to develop only to the point of producing nonviable liver lesions, were not immune to a subsequent challenge infection. This experiment, however, did not include a positive control group and a number of the experimental animals died of a ‘mucoid enteritis-like’ syndrome, and hence, the authors indicate that they could not exclude immunosuppression in the other experimental animals interfering with their ability to develop a protective immune response after the initial exposure to eggs.

Results of experimental investigations on immunity to superinfection in domestic animals confirm the development of immunity to re-infection after an initial experimental infection. Penfold (41,42) undertook a series of small but well-controlled experiments providing evidence that cattle which had received a large experimental infection with *T. saginata* (400 000 eggs) were immune to a subsequent challenge infection for as long as 18 months after the initial infection. Urquhart (43) found that young calves raised in conditions where many animals became naturally exposed to *T. saginata* eggs within the first day after birth were resistant to a later experimental challenge infection. This was interpreted as being because of the animals having previous exposure to the parasite; however, the data were confounded by a proportion of the animals that were resistant to the challenge infection showing no sign of pre-existing infection. Convincing evidence that cattle exposed to *T. saginata* eggs become resistant to a challenge infection was provided by Gallie and Sewell (44) whose data are discussed in more detail below.

Sweatman (45) showed conclusively that sheep which had been given an initial experimental infection with *T. hydatigena* were solidly immune to a challenge infection with the parasite given 7 weeks after the initial infection. Injection of activated *T. hydatigena* oncospheres, and the subsequent growth of the parasites around the site of injection, leads to immunity to a subsequent oral infection (46). Heath *et al.* (47) used a combination of oral infection and injections of activated oncospheres and demonstrated that this procedure resulted in total resistance of sheep to challenge infection with homologous parasites: *T. hydatigena, T. ovis or Echinococcus granulosus*.

Gemmell (48) undertook experiments designed to determine what minimum number of parasites in a primary infection were required to induce immunity against subsequent infection. Sheep were dosed with either 10, 100 or 1000 eggs of *T. hydatigena* or *T. ovis* or were given ten consecutive doses of either 10 or 100 eggs at weekly intervals. The intention was to compare the number of parasites seen at necropsy in those given multiple divided doses and to judge from that number how many doses successfully established as viable parasites prior to the animals becoming immune and accumulating no further cysts. The results have been interpreted to suggest that as few as 10 eggs may be sufficient to achieve immunity to subsequent infections (48–50). However, the reliability of even a single infection with 10, 100 or 1000 parasites to produce any detectable infection (viable or otherwise) in the experimental animals was so poor that it makes interpretation of the data difficult. Gemmell and Johnson (50) provide statistical analysis of the data described in Gemmell’s earlier paper (48) and the results were mixed; sheep receiving 1000 eggs of *T. hydatigena* in a single dose had numbers of parasites that were not significantly different from the numbers seen in sheep given ten doses of 100 eggs.

Flisser *et al.* (51) attempted to summarize the results of many publications using an index based on the proportion of the eggs in a challenge infection that were infective and comparing ‘normal’ and ‘immune’ hosts. These data included studies on taeniid species infecting rodents, rabbits, pigs, sheep and cattle. Eggs established more efficiently in normal hosts, however, it is unclear what situations were amalgamated under the term ‘immune’ and hence it is difficult to interpret the tabulated data.

### Is immunity maintained only with continued exposure to eggs?

One of the commonly accepted beliefs arising from studies on resistance to superinfection with taeniid cestodes is that animals infected early in life gain life-long immunity provided they are frequently re-exposed to parasite eggs. Much of the data which supports this hypothesis comes from failed attempts to experimentally infect animals derived from a population of animals some of which have a naturally acquired infection. These data are unreliable and evidence to support the existence of prior infection in resistant animals may not correlate with resistance to the challenge infection (43). It was mentioned earlier that rats may develop immunity to a challenge infection even in circumstances where the initial exposure to eggs did not result in the establishment of any viable larvae. A similar phenomenon was also shown to be true for *T. hydatigena* or *T. ovis* in sheep (52). Hence, a failure to detect viable parasites in animals raised in conditions likely to lead to natural exposure to the parasite, and which were found to be resistant to an experimental infection without evidence confirming the presence of a pre-existing infection, cannot be taken as evidence to reject the hypothesis that immunity in these animals was the result of prior exposure to the parasite. There is anecdotal evidence on the resistance of animals naturally exposed to taeniid cestodes to an experimental infection, but there are few data available to suggest how frequently this exposure needs to be, nor what quantity of eggs is required, to maintain the immune status. Gallie and Sewell (44) investigated these issues in relation to *T. saginata* infection in cattle. Three groups of cattle were used. Two groups received 10 000 eggs at 2–3 days of age. One of these groups also received repeated doses of 500 eggs at weekly intervals for the following 12 months. At 12 months of age, all three groups were given a challenge infection with 50 000 eggs and the animals subjected to necropsy after the challenge infection at a time when it would be possible to distinguish between parasites persisting from earlier infections and the more recent, large challenge infection. The results of the trial are shown in Table 1. There were slightly more parasites in the animals that received weekly exposures to the parasite, but the difference was not statistically significant in comparison with animals only given 10 000 eggs at 2–3 days of age. Those animals that received only the initial exposure to *T. saginata* were highly susceptible to the challenge infection. Of these five animals, two contained mature viable parasites from the initial infection, which clearly did not prevent infection from the challenge at 12 months of age. Those animals that had received infections at 2–3 days of age, as well as further weekly infections over the 12-month period, were completely resistant to the establishment of any viable parasites from the challenge infection. Four of the five animals were completely resistant to the challenge infection, while the remaining animal developed only eight nonviable lesions. These data provide the best available experimental evidence to support the notion that a trickle exposure to eggs can maintain immunity. In the group given the trickle infection, 2 of the 5 animals had no viable, mature parasites present at the time of necropsy and yet they were resistant to the challenge infection, confirming data from *T. taeniaeformis* and *T. hydatigena*, that the continued presence of viable metacestodes is not requisite for the establishment of a state of immunity to re-infection.

**Table 1:** Number of cysts found in calves following challenge with 50 000 eggs of *Taenia saginata*. Reproduced from Gallie and Sewell (44)

		No. of cysts in carcase		
		Prechallenge cysts		
Group No.	Infection schedule	Alive	Dead	Cysts derived from challenge infection
1	Repeated infections from 2 to 3 days old until challenge at 12 months.	20 ± 27	7 ± 6	8 dead cysts in one animal only.
2	Single infection at 2–3 days old plus challenge at 12 months.	2 ± 3	5 ± 5	6080 ± 2969
3	Challenge at 12 months only.	–	–	8177 ± 3015

The figures shown are means ± standard deviations. The difference between the numbers of the older cysts in Groups 1 and 2 or between the number of cysts derived from the challenge infection in Groups 2 and 3 were not statistically significant.

The following issues concerning concomitant immunity in the intermediate hosts of taeniid cestode parasites can be regarded as having been clearly established:

1. Following a relatively large, experimental infection, hosts develop a high level of resistance to re-infection.
2. The development of immunity relates to the exposure of the host to early developing parasites.
3. Induction of immunity is not dependent upon the establishment of viable, mature parasites from the initial exposure to parasite eggs.
4. Immunity wanes irrespective of the continued presence of mature metacestodes.
5. Immunity can be maintained experimentally by repeated, frequent re-exposure to parasite eggs.

The following issues are not well established:

1. The minimum infecting dose of eggs required to induce protective immunity.
2. The duration of immunity to re-infection in the absence of further exposure to eggs.
3. The minimum number of eggs required, or the frequency required, for repeated egg exposure to maintain protection.
4. The extent to which the level of exposure to taeniid cestode eggs that occurs in normal endemic situations is sufficient to develop immunity and serve to prevent the accumulation of further infection.
5. Whether ‘early’ and ‘late’ immunity are associated with different parasite antigens.

An understanding of the dynamics of immunity to re-infection with taeniid cestodes would aid in making accurate predictions of what could be expected to occur in various endemic situations.

### Evidence from age-related differences in infection prevalence and intensity

There are relatively few studies of the intensity of taeniid cestode infections in naturally exposed animals that are not compromised by the methodologies used. Gemmell (53) examined damage to livers caused by *T. hydatigena* in sheep of different ages and stated in conclusion “it is not unreasonable to suggest that in the field the primary infestation of cysticerci results in an absolute or at least a relative immunity against later infestations of *T. hydatigena* in New Zealand sheep”. The study assessed liver damage and did not enumerate the number of parasites in each animal. Surveys of *E. granulosus* infection in domestic animals have identified an increasing prevalence in successive age classes (54–58). However, this cannot in itself be used as an indication of a lack of immunity. Some studies either did not undertake detailed examination of the organs affected (59) or it is not entirely clear what procedures were used to identify hydatid cysts (56,57). There are substantial difficulties in identifying small hydatid cysts which can limit detection in younger animals, depending on the procedures used (59,60). However some studies have been carried out which could have been expected to identify small, younger hydatid cysts accurately, and these studies have identified an increase in the prevalence of infection (55,58,61). More importantly from the point of view of immunity affecting disease epidemiology is that Lamar *et al.* (58) and Dueger *et al.* (61) found that sheep in endemic areas had an increasing *intensity* of infection with increasing age, with animals 1–2 years of age having on average about two cysts while animals ≥5 years of age had about 8–10 cysts. This is not evidence against the possibility that immunity is limiting the accumulation of new cysts in re-exposed animals, but it does provide evidence to suggest that immunity does not prevent the establishment of new cysts in already infected animals in a natural field situation.

## Concluding remarks

Although much has been discovered in relation to immunity to taeniid cestode infections, there is clearly much still to be learnt. Aspects such as the duration of immunity following initial infections with different numbers of parasites, what frequency and what minimum number of parasites is required to maintain immunity, and whether immunity to vaccines is boosted by natural exposure to oncospheres, are important issues for future research. Such information would be valuable to assist with preparation of accurate models of disease transmission, particularly as they apply to the practical use of vaccines.

A number of other issues pertaining to immunity to taeniid cestode infections are the subject of conflicting data or opinions, and warrant careful consideration. These include, among others, the strength of the evidence to support the existence of host protective immune responses in definitive hosts, the immunogenicity of oncosphere penetration gland secretions and the role of these glands in assisting oncospheres penetrate the host intestine, and the usefulness of *Taenia crassiceps* as a model for *Taenia solium* vaccine development and the S3Pvac vaccine developed using this model.
